# Multi - dimensional mechanism analysis of *Choerospondias axillaris* (Roxb.) Burtt et Hill in treating kidney stones: network pharmacology, molecular docking and *in vitro* experimental verification

**DOI:** 10.3389/fphar.2025.1501386

**Published:** 2025-04-16

**Authors:** Meiqi Qiu, Senhua Li, Yu Zhang, Jiaoxia Yan, Shiting Qin, Xijing Yin, Yujun Li, Chunhui Zeng, Ke Yang

**Affiliations:** College of Pharmacy, Guangxi University of Chinese Medicine, Nanning, Guangxi, China

**Keywords:** Choerospondias axillaris (Roxb.) Burtt et Hill, kidney stones, network pharmacology, molecular docking, calcium oxalate crystals, NLRP3 inflammasome

## Abstract

**Ethnopharmacological relevance:**

*Choerospondias axillaris* **(Roxb.) Burtt et Hill** (CA) is a medicinal and edible plant fruit with national characteristics in China. CA is commonly used in folk medicine for treating kidney diseases, heart diseases, and calming the mind. Modern pharmacological studies have demonstrated that CA exhibits effects such as protection against renal injury, antioxidation, anti-inflammation, and calcium antagonism.

**Objective:**

This study aimed to explore the mechanism of CA in treating kidney stones through network pharmacology, molecular docking, and ***in vitro*** experiments.

**Methods:**

Network pharmacology was employed to screen the active components of CA. The targets of these active components and disease-related targets were predicted, and potential targets were obtained by taking the intersection of the two sets. The potential targets were then used to construct a Protein-Protein Interaction (PPI) network and the core targets were screened out. Gene Ontology (GO) and Kyoto Encyclopedia of Genes and Genomes (KEGG) pathway enrichment analyses were conducted on the potential targets. A Drug-Component-Target-Pathway network was constructed. Molecular docking was carried out between key active ingredients and their corresponding core targets. Through the above methods, the mechanism of action of CA in treating kidney stones was preliminarily predicted. In addition, the effect of CA on the growth of Calcium Oxalate (CaOx) crystal was investigated in two-dimensional CaOx agar gel system. The protective effect of CA and its mechanism were explored in the model of Human Kidney Cortex (HKC) cell injury induced by Calcium Oxalate Monohydrate (COM) crystals.

**Results:**

Through network pharmacology analysis, 9 active ingredients were obtained, namely, (−)-taxifolin, naringenin, (−)-catechin, quercetin, bis [(2S)-2-ethylhexyl] benzene-1,2- dicarboxylate, (2R)-5,7-dihydroxy-2-(4-hydroxyphenyl) chroman-4- one, beta-sitosterol, ellagic acid, kaempferol. There were 272 protein targets for the active ingredients and 3,525 for diseases. After intersecting the two sets, 187 potential targets were identified. PPI network analysis revealed that the top five core targets were AKT1, IL6, TNF, TP53, and IL-1β. GO analysis indicated that the treatment of kidney stones with CA was involved in biological processes like the response to oxidative stress and regulation of inflammatory response. KEGG prediction suggested that the treatment of kidney stones with CA was closely associated with signaling pathways such as NF-κB and MAPK. Molecular docking results demonstrated that five key active ingredients (quercetin, kaempferol, (−)-catechin, β-sitosterol and naringenin) exhibited good binding ability with their corresponding core targets. The results of two-dimensional CaOx agar gel system showed that the CA-L group significantly decreased the aggregation of COM crystals. In the CA-M and CA-H groups, the crystals mainly existed in the form of Calcium Oxalate Dihydrate (COD), which was readily excreted with urine, causing minimal damage to renal epithelial cells. Moreover, the crystal surface area was significantly smaller compared that of the model group. CA could protect cells damaged by COM crystals by increasing SOD activity, reducing ROS levels, and decreasing lactate dehydrogenase (LDH) leakage. Simultaneously, CA downregulated the expression of inflammatory proteins such as NLRP3, Caspase-1, IL-1β, as well as the expression of OPN protein, which promotes crystal adhesion.

**Conclusion:**

CA can attenuate the damage and adhesion of COM crystals to cells through multiple mechanisms. These include enhancing the cellular antioxidant capacity, regulating the activation of the NLRP3 inflammasome, reducing the expression of the crystal adhesion protein OPN, and preventing the further aggregation or mineralization of CaOx crystals. Thus, CA achieves the objective of treating kidney stones.

## 1 Introduction

Kidney stones are a prevalent chronic urinary system disease, characterized by a high incidence and recurrence rate. Surveys indicate that the global incidence of kidney stones has reached 14.8% and is still on the rise (Romero et al., 2010). Moreover, the recurrence rate can be as high as 50% within 5 years after the initial occurrence (Tanaka et al., 2021). With the changing eating habits and lifestyles of people, kidney stones not only have the potential to progress into more serious lifelong kidney disease, but may also be accompanied by life - threatening complications such as hypertension and myocardial infarction (Alexander et al., 2014; Khan et al., 2016). CaOx stones are the most common type among kidney stones. Currently, the main treatment approach is surgical removal. However, due to its high recurrence rate and occurrence of serious complications, this method has increased the global clinical and economic burden. Consequently, there is an urgent need to identify an effective and cost - effective drug for the treatment of CaOx stones.

Traditional Chinese medicine offers notable advantages, such as remarkable effect and minimal toxic and side effects, in the treatment of kidney stones. CA is the dried and mature fruit of *Choerospondias axillaris* **(Roxb.) Burtt et Hill**. The mature drupe is oval or obovate-oval in shape, with five small holes at the apex, 2–3 cm in length and 1.4–2 cm in diameter. The surface is dark brown or tan, slightly shiny, with irregular wrinkles, and a fruit stalk scar at the base. It is commonly known by various names, including “wuyanguo,” “Guangzao,” “renmianzi,” “shanzaozi,” etc (Wang et al., 2023). CA is a national characteristic medicine that falls under the category of substances with dual roles in medicine and food (Xiong et al., 2023; Ye et al., 2024). It is an endemic plant in Southeast Asia and is widely distributed in the southern regions of the Yangtze River in China (Wang et al., 2023). Since the 2000 edition of the Chinese Pharmacopoeia, CA has been included in it (Editorial Board of Chinese Pharmacopoeia Committee, 2020). It tastes sweet and sour, with a neutral property. It has functions such as promoting qi and blood circulation, nourishing heart and calming the mind, eliminating stagnation and detoxifying. It is mainly used to treat qi stagnation and blood stasis, chest pain, palpitation and shortness of breath, and restlessness (Editorial Board of Chinese Pharmacopoeia Committee, 2020). It has a long history of medicinal use and is widely applied. The ancient book “Jingzhu Bencao” records: “Guangzao is warm in nature and sour in taste, It clears heart heat, relieves restlessness, alleviates pain, and treats heart disease (Wang et al., 2023). It has often been used to treat various diseases, including heart disease, calming nerves, relieving hangovers, indigestion, bronchitis, and neurasthenia (Wang et al., 2023). Modern studies have revealed that its main components include phenolic acids (such as protocatechuic acid, gallic acid, ellagic acid, etc.), flavonoids (quercetin, kaempferol, dihydroquercetin, etc.) polysaccharides, and volatile oils (Li et al., 2008; Zhou et al., 2017). Its pharmacological effects mainly encompass the protection of acute renal ischemia-reperfusion injury, antioxidation, anti-inflammatory, calcium antagonism, antibacterial, anti-arrhythmia, anti-coagulation, and immunity enhancement properties (Li et al., 2008; Wang et al., 2023). The folk application survey has found that CA exhibits a favorable therapeutic effect on kidney stones. Meanwhile, previous research of this project showed that CA can inhibit the formation of CaOx stones by influencing the autophagy reaction in mice (Yang et al., 2010; Yang et al., 2022). Nevertheless, the specific mechanism by which CA treats kidney stones remains unclear.

Network pharmacology is a novel discipline that is founded on system biology theory, biological system network analysis, and multi-target drug molecules through design specific signal node selection. Natural drugs often have drawbacks such as complex components, multiple targets, and unclear mechanisms of action. Consequently, in this study, network pharmacology was employed to screen the potential targets of CA in the treatment of kidney stones and predict its action mechanism. Molecular docking was utilized to evaluate the binding ability of CA’s active components to the targets, and *in vitro* experiments were conducted for verification. The aim of this study was to preliminarily elucidate the mechanism by which CA treats calcium oxalate kidney stones, thereby laying a solid foundation and providing a theoretical framework for further in - depth research on CA.

## 2 Materials and methods

### 2.1 Reagents and chemicals

Agar powder, mixture of penicillin and streptomycin, MTT, DMSO, SDS were procured from Beijing Soleibao Technology Co., Ltd. Anhydrous calcium chloride was purchased from Shanghai Super Chemical Co., Ltd. Sodium oxalate was obtained from Tianjin Kemiou Chemical Reagent Co., Ltd. Fetal bovine serum was sourced from Zhejiang Tianhang Biotechnology Co., Ltd. DMSO was acquired from Tianjin Damao Chemical Reagent Factory. The whole protein extraction kit was purchased from Jiangsu Kaiji Biotechnology Co., Ltd. The active oxygen detection kit was obtained from Shanghai Beyotime Biotechnology Co., Ltd. The LDH kit and SOD kit were purchased from Nanjing Jiancheng Bioengineering Institute. The 5 × protein-free rapid blocking solution, ultrasensitive chemiluminescence detection kit, and 10% PAGE gel rapid preparation kit were all purchased from Shanghai Yamei Biomedical Technology Co., Ltd. The primary antibody dilution and secondary antibody dilution were purchased from Shanghai Beyotime Biotechnology Co., Ltd. The BCA protein kit was purchased from Boster Bioengineering Co., Ltd. The 1,640 medium purchased from Gibco (USA). Rainbow protein Marker (#01057501, thermoFisher), GAPDH antibody (#GR217575-64) and Osteopontin antibody (#GR196305-14) were all purchased from Abcam; The NLRP3 antibody (#87p812), Caspase-1 antibody (#23m5315) and IL-1β antibody (#63h9328) were all obtained from Affinity Biosciences. The horseradish enzyme-labeled goat anti-rabbit IgG antibody was purchased from proteintech Group.

### 2.2 Network pharmacology

#### 2.2.1 Screening of active components and their targets of CA

In the TCMSP database (https://old.tcmsp-e.com/tcmsp.php), “Wuyanguo,” “Nansuanzao” and “Guangzao” were used as the search terms to search for the chemical constituents of CA. The active ingredients were screened according to oral bioavailability (OB)≥30% and drug-likeness (DL)≥0.18, and the protein targets related to active ingredients were collected. Gene name was standardized by UniProt database (https://www.uniprot.org/). The active components were searched in the CTD database (https://ctdbase.org/) and gene targets with scores greater than 10 were screened. The targets of the two databases were taken and collected, and the.

“Drug-Component-Target” network was constructed using Cytoscape 3.7.1 software.

#### 2.2.2 Screening of potential targets for the treatment of kidney stones with CA

The targets related to kidney stones disease retrieved from DisGeNET database (https://ctdbase.org/), OMIM database (https://www.omim.org/) and Genecards database (http://www.liuxiaoyuyuan.cn/) were integrated and deduplicated to obtain disease targets. Venny2.1.0 was mapped to the target of CA to obtain the potential target of CA in the treatment of kidney stones.

#### 2.2.3 PPI network construction of potential targets and screening of core targets

To explore the interactions between potential targets, the potential targets for the treatment of kidney stones disease by CA were input into the STRING database (https://string-db.org/). The species were selected as “Homosapiens,” and the interaction threshold default system was set to score greater than 0.4. The file was downloaded in TVS format and imported into Cytoscape3.7.1 software for PPI network visualization, and the plug-in CytoNCA was used to analyze and screen its core targets.

#### 2.2.4 GO and KEGG enrichment analysis

With the help of Metascape platform, GO and KEGG enrichment analysis were performed on the potential targets of CA in the treatment of kidney stones. With human genes as the background, set P1.5. Based on the LogP value, the top 15 biological processes (BP), cellular components (CC), molecular functions (MF) and the top 20 KEGG signaling pathways were screened. The results were visualized and analyzed by using the online platform (https://www.bioinformatics.com.cn/), and the “Drug-Component-Target-Pathway” network was constructed by using Cytoscape 3.7.1 software, and the key active components of CA were screened according to the Degree value.

### 2.3 Molecular docking

The two-dimensional format of small molecules of key active components of CA was obtained from PubChem and converted into mol2 format by PyMOL. The corresponding target files were obtained in the PDB database (http://www1.rcsb.org/), and the original ligands and water molecules were deleted in PyMOL. The small molecules and targets were imported into AutoDockTools 1.5.6 software for molecular docking, and PyMOL software was used for visual analysis.

### 2.4 Experimental verification

#### 2.4.1 Preparation of drugs

CA was identified as the dried fruit core of *C. axillaris* **(Roxb.) Burtt et Hill** (Herbarium number: 01981684) by Associate Professor Guo Min from Guangxi University of Chinese Medicine. Specifically, 10.29 kg of crude powder was taken. Then, 60 L of distilled water was added, and the mixture was soaked for 30 min. Subsequently, it was heated and extracted for 2 h, after which it was filtered. Next, 50 L of distilled water was added to the drug residue, and the residue was heated and extracted for 1 h followed by another filtration. Finally, the two filtrates were combined and concentrated to 1.83 kg, thereby obtaining the aqueous extract of CA.

#### 2.4.2 Construction of two-dimensional calcium oxalate agar gel system

A 1% agar solution containing 0.01 mol/L sodium oxalate was poured into the culture dish to serve as the solvent control group (UP group) (Li et al., 2015). Meanwhile, 1% agar solution containing 0.01 mol/L sodium oxalate and 25.0, 12.5, 6.25 mg crude drug/mL CA water extract were respectively poured into separate culture dish, which were designated as the high, medium and low dose groups (CA-H, CA-M, CA-L groups). After cooling and solidification, gels containing oxalate ions were formed. A round hole was made in the center of each gel. Every day, a fixed amount of 2.0 mol/L calcium chloride solution was added to ensure that the final concentrations of calcium ion and oxalate ion were consistent. The culture dishes were then sealed in an electric thermostatic incubator (Shanghai Yuejin Medical Device Factory, 500-BS-11, China) at 37°C. The experimental duration was set to 7 days.

#### 2.4.3 Study on diffusion surface and crystal morphology of the system

On the 7th day, the two-dimensional calcium oxalate agar gel system was photographed and recorded. Approximately 0.25 cm^2^ CaOx crystals were taken from different crystal diffusion surfaces on placed on glass slides. Then, 100 μL of ultrapure water was added, and coverslips were placed on top. The CaOx crystals on different diffusion surfaces of the system were using a biomicroscope (OLYMPUS, BX53F, Japan).

#### 2.4.4 Fourier transform infrared spectroscopy study

After drying the agar gel at 60°C, the CaOx crystal was ground into powder in agate mortar. Subsequently, the powder was mixed with potassium bromide (KBr) containing 0.2% potassium thiocyanate (KSCN). The mixture was then pressed under the condition of 60 MPa and 1 min. The resulting sample was characterized by FT-IR using Fourier transform infrared spectrometer (Thermofisher, Nicolet 6700, United States).

#### 2.4.5 X-ray diffraction studies

The CaOx crystal powder was characterized by X-ray diffraction (XRD) using a Bruker AXS instrument (Bruker D8Advance, Germany). The XRD test conditions were as follow: Cu target Kα radiation, a graphite bent crystal monochromator, 40 KV accelerating, 100 mA, tube current, slits with a divergence slit (DS) of 1°, a receiving slit (RS) of 0.15 mm, and a scatter slit (SS) of 1°. The scanning speed was 10°·min^−1^, the scanning range was from 10° to 60°, and the step width was 0.02°.

#### 2.4.6 Establishment of HKC cell model of COM crystal-damage and the treatment with CA

HKC cells (obtained from the Scientific Experimental Center of Guangxi University of Chinese Medicine, Nanning, China) were cultured in 1,640 complete medium (supplemented with 10% fetal bovine serum and 1% penicillin-streptomycin mixture) in an incubator maintained at 37°C, 5% CO_2_ incubator for 24 h. The cells were then divided into a control group, a model group, and CA groups (at concentrations of 500 μg/mL, 200 μg/mL, 100 μg/mL, and 50 μg/mL). The control group received no treatment. The model group was treated with 100 μg/mL COM crystal solution. For the CA group, 100 μg/mL COM crystal solution and corresponding concentration of CA water extract were added to the cells. The cells were then cultured in the 37°C, 5% CO_2_ incubator for 24 h.

#### 2.4.7 Detection of LDH leakage in cells

The leakage of LDH in the supernatant was detected by microplate method. HKC cells were seeded in 96 well plates according to the above-mentioned procedure. After the culture period ended, the cell supernatant was collected and centrifuged at 4000 rpm for 10 min. Then, 5 μL supernatant was taken. Subsequently, the corresponding reagents were added in strict accordance with the instructions of the LDH kit. Finally, the absorbance value of each well was measured at the wavelength of 450 nm using a microplate reader (BioTek Instruments, Synergy H1, United States).

#### 2.4.8 Detection of cell viability

MTT colorimetry was used to detect the cell survival rate. HKC cells were treated in 96-well plates as described above. Once the culture time elapsed, the cells supernatant was discarded. Then, 100 μL of a 10% MTT solution was added to each well, and the cells were incubated in a 37°C, 5%CO_2_ incubator for 4 h. After the incubation, the supernatant was removed, and 150 μL DMSO was added to each well. The plates were then placed on a microplate shaker at room temperature for 10 min to completely dissolve the formazan crystals. The absorbance value was measured at a wavelength of 570 nm using a microplate reader.

#### 2.4.9 Detection of SOD enzyme activity

The activity of superoxide dismutase (SOD) was detected using WST-1 method. HKC cells were seeded in 6-well plates following the above protocol. After the culture period, the cell supernatant was discarded, and the protein was extracted and its concentration was determined according to the method in Western blot analysis. The supernatant was dilute, and 20 μL of the diluted supernatant was used for the operation in accordance with the instructions of the SOD kit. The reading of the microplate reader at 450 nm was recorded. The SOD enzyme activity was calculated after ensuring that the SOD inhibition rate of cells in each group ranged from 40% to 60%.

#### 2.4.10 Detection of intracellular ROS content

HKC cells were seeded in 6-well plates according to the above method. After the culture time, the cell supernatant was discarded. Then, CAFH-DA was added, and the cells were incubated in a 5% CO_2_ incubator at 37°C for 20 min. The supernatant was removed, and the medium was used to wash the cells for 3 times. The Petri dish was placed under a fluorescence microscope (Leica, DMI8, Germany). After the images were collected, the fluorescence intensity was analyzed using ImageJ software.

#### 2.4.11 Western blot analysis

The cells were lysed with pre-cooled cell protein lysate. The supernatant was then centrifuged at 15,000 rpm at 4°C for 20 min to obtain protein samples. The protein concentration was detected using the BCA method. The protein sample was mixed with the protein loading buffer and denatured at 100°C for 10 min after that, it was cooled at room temperature and stored in a −20°C refrigerator. The sample was separated on an SDS-PAGE gel and transferred to a PVDF membrane. The menbrane was blocked in a protein-free rapid blocking solution for 30 min and then incubated overnight with primary antibodies at 4°C: GAPDH antibody (1:10,000), anti-NLRP3 (1:1,000), anti-Caspase-1 (1:1,000), anti-IL-1β (1:1,000), anti-OPN (1:1,000). The PVDF membrane was placed in a horseradish enzyme-labeled goat anti-rabbit IgG (1:5,000) secondary antibody working solution and incubated at room temperature for 2 h. After ECL coloration, the Gel Doc 2000 Gel Documentation System was used for imaging, and ImageJ software was used to measure the intensity.

### 2.5 Statistical analysis

Statistical analysis was conducted using SPSS 11.5 statistical software. Measurement data were presented as the mean ± standard deviation ( $\bar{x}$ ±SD). A paired t - test was employed for inter - group comparisons. A significance level of *P* < 0.05 was regarded as statistically significant.

## 3 Results

### 3.1 Active components and targets of CA

**FIGURE 1:**
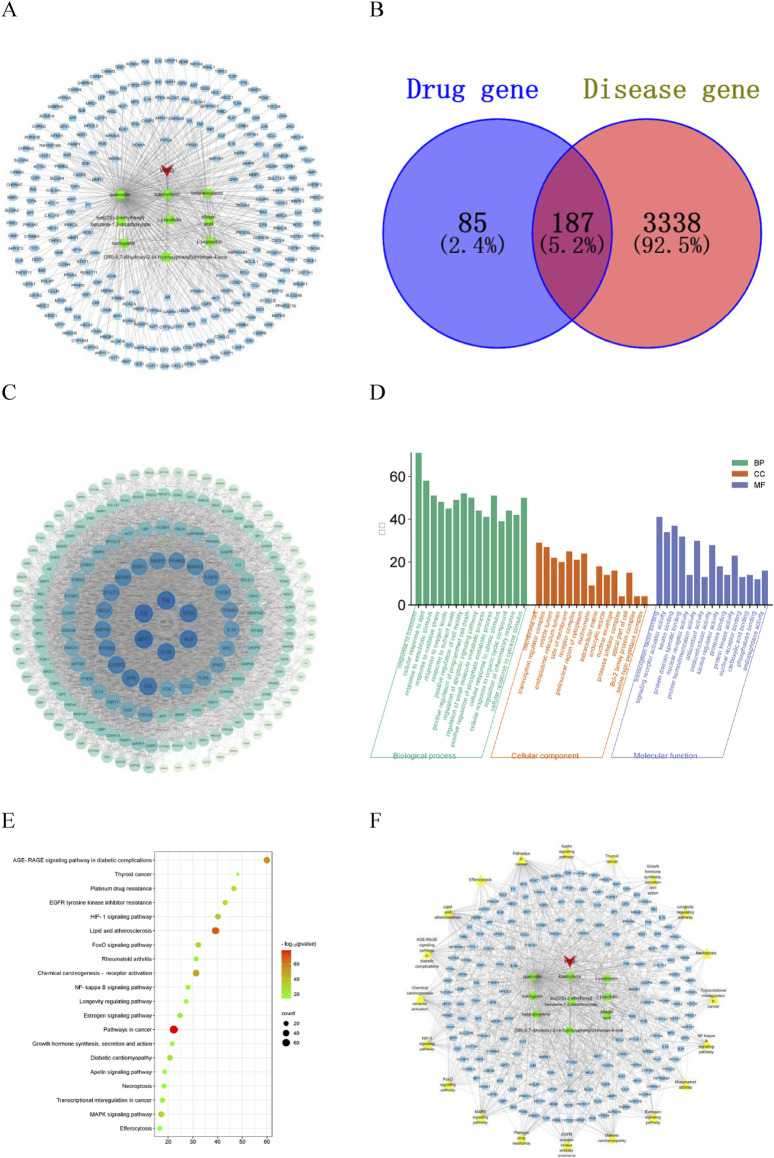
Network pharmacology analysis diagram. **(A)** Drug-Component-Target diagram; **(B)** potential target map; **(C)** PPI diagram of potential targets; **(D)** GO enrichment analysis diagram; **(E)** KEGG enrichment analysis diagram; **(F)** Drug-Component-Target-Pathway diagram.

Nine active components of CA were screened by TCMSP database (Table 1), and 272 targets were obtained by combining UniProt database correction with active component targets obtained by CTD database. Cytoscape 3.7.1 was used to construct the “Drug-Component-Target” network diagram (Figure 1A). In the diagram, red represents the CA, green represents the active ingredient, blue represents the target, and each edge represents the relationship between the target and the target.

**TABLE 1:** Active components of CA.

MOLID	Molecule name	Molecular weight	OB%	DL
MOL001736	(−)-taxifolin	304.27	60.51	0.27
MOL004328	naringenin	272.27	59.29	0.21
MOL000096	(−)-catechin	290.29	49.68	0.24
MOL000098	quercetin	302.25	46.43	0.28
MOL001490	bis [(2S)-2-ethylhexyl]benzene-1,2-dicarboxylate	390.62	43.59	0.35
MOL001002	ellagicacid	302.2	43.06	0.43
MOL001040	(2R)-5,7-dihydroxy-2-(4-hydroxyphenyl)chroman-4-one	272.27	42.36	0.21
MOL000422	kaempferol	286.25	41.88	0.24
MOL000358	beta-sitosterol	414.79	36.91	0.75

### 3.2 Potential targets for the treatment of kidney stones by CA

A total of 3,525 kidney stones disease targets were obtained from the GeneCards, OMIM and DisGeNET databases, of which 187 were potential targets for the treatment of kidney stones, accounting for 69% of the active ingredient targets (Figure 1B). The prediction results show that the CA has great potential for the treatment of kidney stones.

### 3.3 PPI network construction of potential targets and screening of core targets

The potential targets were input into the STRING database to construct a protein-protein interaction network. The network topology analysis and PPI network visualization were performed using the function of “Network Analyzer” in Cytoscape3.7.1 software (Figure 1C). The circular nodes in the figure represent different gene proteins, and the node connection represents the interaction between proteins. The size and color depth of the circular nodes represent the degree value. The larger the node and the darker the color, the greater the degree value, and the greater the degree value indicates that the protein plays a greater role in the network. The CytoNCA plug-in was inserted to screen the targets, and Degree, Betweenness Centrality and Closeness Centrality exceeded their medians as the core targets for the treatment of kidney stones. Considering the degree and closeness between the targets, the first five core targets were selected as AKT1, IL6, TNF, TP53 and IL-1β.

### 3.4 GO biological function enrichment and KEGG signaling pathway analysis

The potential targets were analyzed on the Metascape platform and visualized using the online platform. The results of GO enrichment analysis showed that there were 2,188 items related to the treatment of kidney stones by CA, including 1869 biological process items, which mainly involved response to oxidative stress, and regulation of inflammatory response, etc. There were 114 items of cell components, mainly involving membrane raft, receptor complex, extracellular matrix, etc. There were 205 molecular functions, mainly involving transcription factor binding, oxidoreductase activity, protein domain specific binding, antioxidant activity, etc. According to the size of P value, the significance of enrichment was judged, and the top 15 GO enrichment analysis of significance was selected for visualization (Figure 1D). The results showed that the CA may play a role in the treatment of kidney stones by regulating the response of cell to oxidative stress, antioxidant activity and inflammatory response. The results of KEGG enrichment analysis showed that a total of 205 pathways related to the treatment of kidney stones by CA were obtained. The main pathways involved were FoxO signaling pathway, NF-κB signaling pathway, MAPK signaling pathway and other signaling pathways. The top 20 of the cluster analysis were visualized (Figure 1E), and the P value represented the bubble color.

### 3.5 “Drug-Component-Target-Pathway” network construction and screening of key components

Nine active ingredients, 187 potential targets and 20 pathways for the treatment of kidney stones were integrated into Cytoscape 3.7.1 software to construct a network diagram (Figure 1F). In the diagram, red represents the drug, green represents the active ingredient, blue represents the target, yellow represents the pathway, and the node connection represents the relationship between the two. The first five Dgree values of the network topology analysis were considered to be key components of the treatment of kidney stones (Table 2). The prediction results show that the CA may play a role in the treatment of kidney stones through multi-component, multi-target and multi-pathway.

**TABLE 2:** The key components of CA in the treatment of kidney stones.

ID	Name	Degree	Betweenness centrality	Closeness centralit
MOL000098	quercetin	228	0.60033485	0.74825175
MOL000422	kaempferol	57	0.05467416	0.41634241
MOL000096	(−)-catechin	44	0.05107955	0.40530303
MOL000358	beta-sitosterol	37	0.05229597	0.39338235
MOL004328	naringenin	34	0.055528	0.38768116

### 3.6 Molecular docking results

**FIGURE 2:**
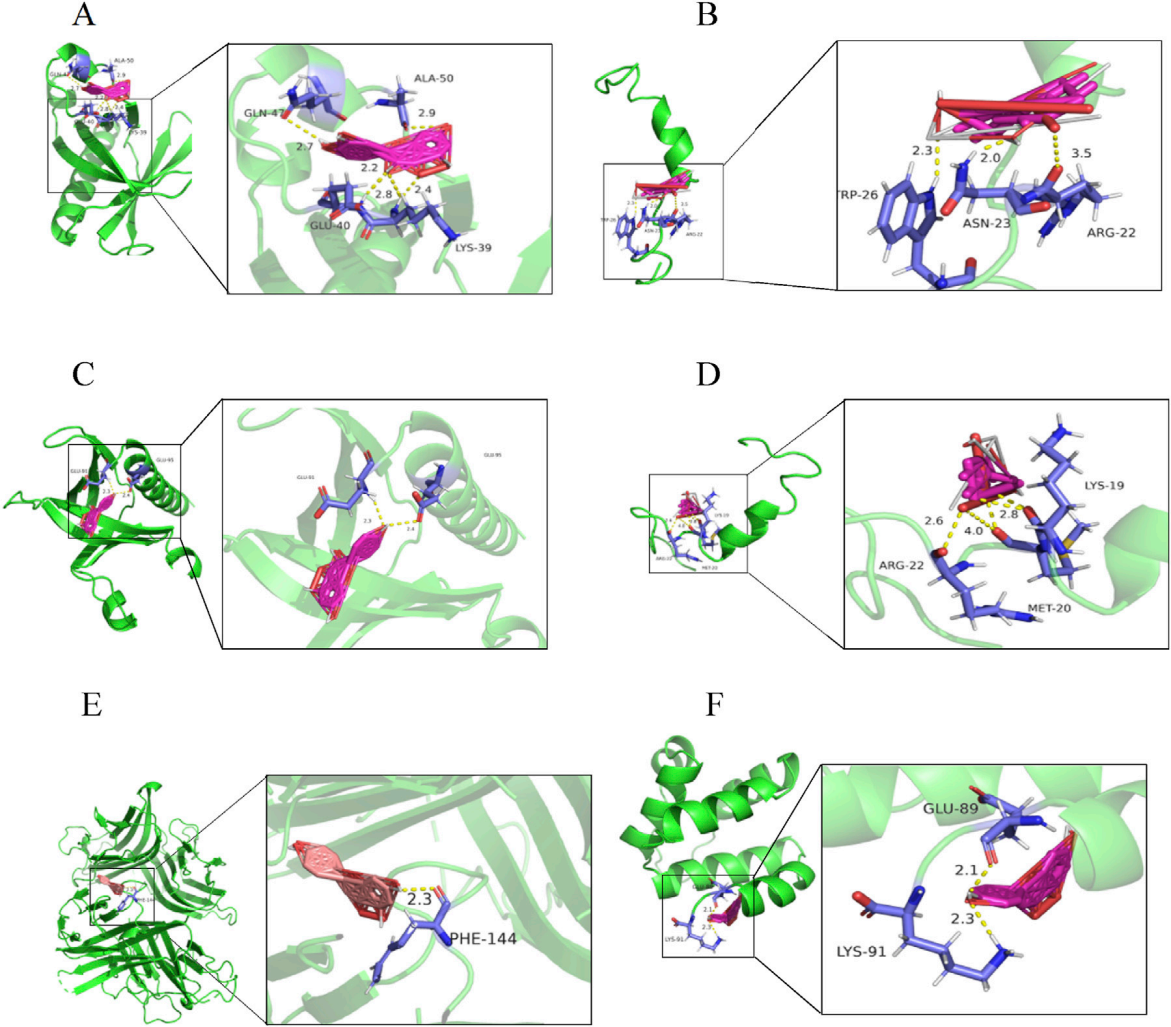
Molecular docking diagram. **(A)** quercetin-AKT1; **(B)** quercetin-TP53; **(C)** kaempferol-AKT1; **(D)** kaempferol-TP53; **(E)** naringenin-TNF; **(F)** naringenin-NLRP3.

The top 5 targets (AKT1, IL6, TNF, TP53, IL-1β) and NLRP3 inflammasome-related targets (NLRP3, Caspase-1) in the PPI network were selected for molecular docking with key components (quercetin, kaempferol, (−) -catechin, beta-sitosterol, naringenin) to further determine the effectiveness of CA in the treatment of kidney stones. The results of molecular docking showed that it had good binding affinity with AKT1, NLRP3, TP53 and TNF (Figure 2; Table 3). It was clear that these compounds were the key components for the treatment of kidney stones, and further verified the mechanism of the treatment of kidney stones.

**TABLE 3:** Molecular docking binding energy of key components of CA and corresponding targets.

Ingredient	Targetname	PDBID	Bindingenergy (kcal/mol)
quercetin	AKT1	1unq	−5.56
	TP53	1q2i	−5.19
kaempferol	AKT1	1unq	−5.51
	TP53	1q2i	−5.94
naringenin	AKT1	1unq	−5.46
	NLRP3	2na9	−6.22
	TP53	1q2i	−6.55
	TNF	2az5	−5.37
beta-sitosterol	AKT1	1unq	−5.59
	TP53	1q2i	−6.01
	TNF	2az5	−5.73
(−)-catechin	AKT1	1unq	−5.12
	NLRP3	2na9	−4.54
	TP53	1q2i	−5.48

### 3.7 CA inhibit the formation and aggregation of COM crystals

**FIGURE 3:**
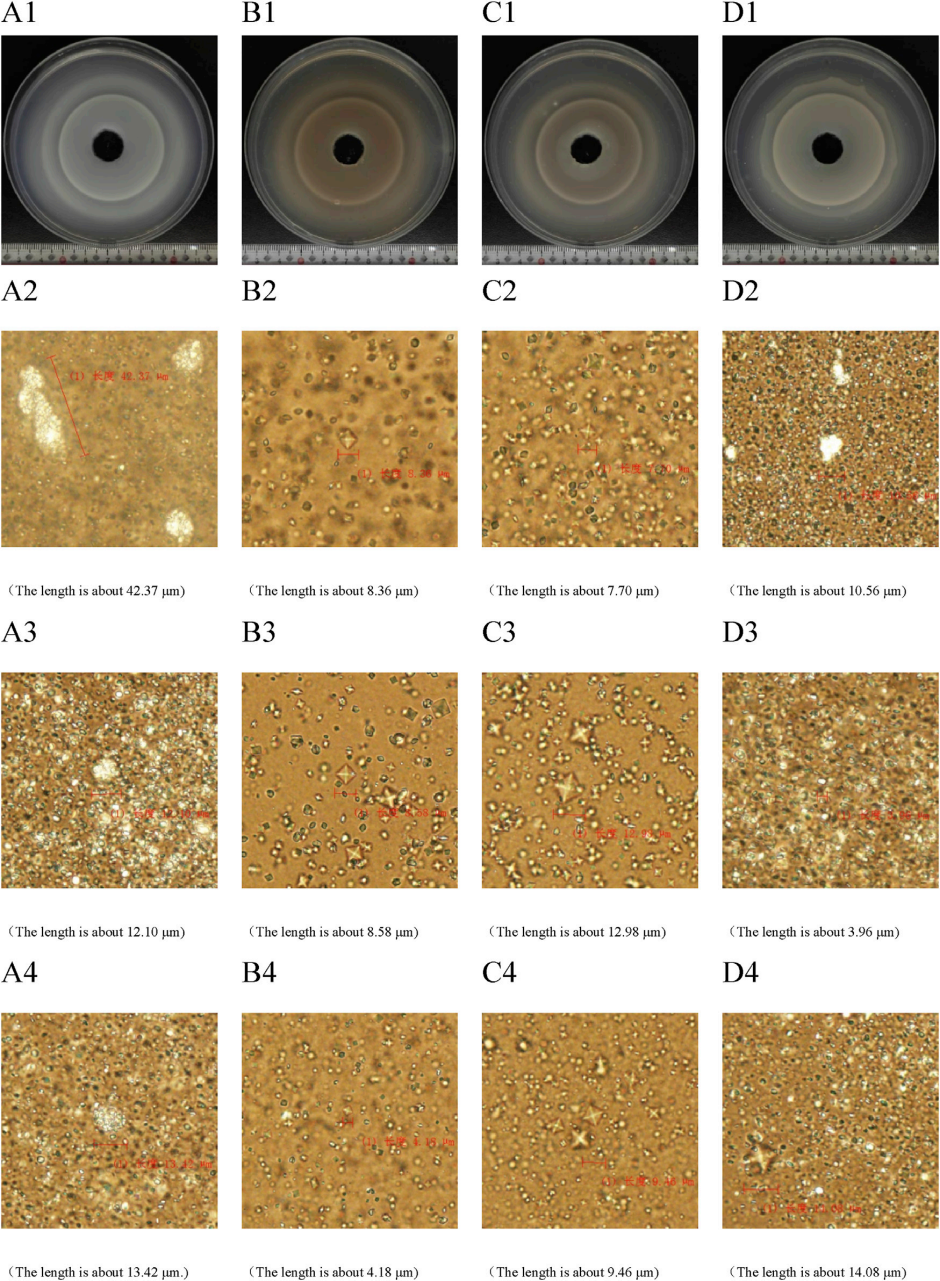
Two-dimensional calcium oxalate agar gel system diagram. **(A)** UP group; **(B)** CA-H group; **(C)** CA-M group; **(D)** CA-L group. **(A1–D1)** periodic ring precipitation diagram of CaOx crystal. **(A2–D2)** The first crystal diffusion surface was characterized by microscope. **(A3–D3)** microscope characterization of the second crystal diffusion surface. **(A4–D4)** microscopic characterization of the third crystal diffusion surface.

In the two-dimensional agar gel disk system (Figures 3A1–D1), the growth of CaOx crystals was characterized by the formation of multiple crystal diffusion surfaces. The farther out the crystal diffusion surface, the lighter its color, indicating a lower formation amount of COM aggregates. In the CA-M group, the first crystal diffusion surface exhibited a distinct two - layer structure, an inner layer and outer layer. This phenomenon indicated that CA-M had a strong inhibitory effect on the diffusion of calcium chloride. It effectively slowed down the diffusion rate of calcium chloride, thereby promoting the premature formation of the crystal diffusion surface. In the CA - L group, the second crystal diffusion surface also showed a two - layer structure. This suggested that CA - L might have inhibited the formation of COM aggregates to a certain extent, but failed to completely prevent the generation of COM crystals. Instead, it promoted the formation of calcium oxalate dihydrate (COD) crystals. These results fully demonstrated that CA could effectively delay the formation, aggregation, and even mineralization processes of COM.

The results of biological microscope characterization (Figures 3A2–D4) showed that in the UP group and the CA - L group, the crystals mainly consisted of a large number of COM crystals in the shape of hemp seeds. In the UP group, three relatively large rod - shaped or spherical COM aggregates were formed. In contrast, the size of the COM aggregates in the CA - L group was significantly smaller. In the CA - H group and the CA - M group, the crystals predominantly formed into a large number of quadrilateral biconical COD crystals. Compared with the model group, the crystal plane of the CA group was significantly reduced. This clearly indicated that CA had the ability to inhibit the formation and aggregation of COM crystals and promote the formation of COD crystals, ultimately resulting in a reduction of the crystal size.

### 3.8 FT-IR characterization analysis

**FIGURE 4:**
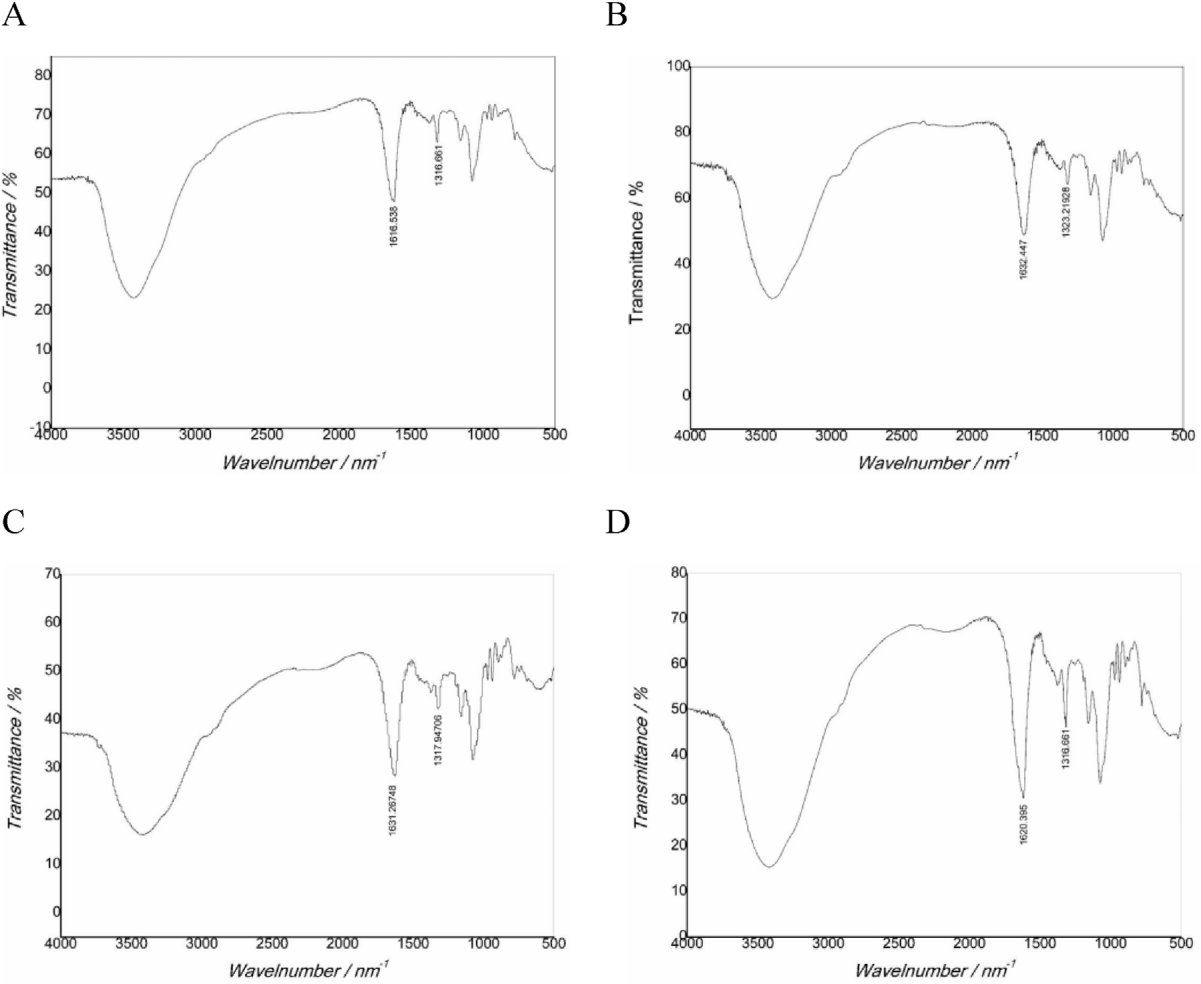
FT-IR spectra of CaOx crystals in two-dimensional calcium oxalate agar gel system. **(A)** UP group; **(B)** CA-H group; **(C)** CA-M group; **(D)** CA-L group.

FT-IR characterization results (Figure 4; Table 4) showed that the main characteristic peaks of the crystals in the gel were basically consistent with the characteristic peaks of CaOx infrared spectrum (Maurice-Estepa et al., 2000; Tan et al., 2003). Regarding pure COM crystals, the asymmetric stretching vibration absorption peak Vas (COO^−^) is located at 1,618 cm^−1^, while the symmetric stretching vibration absorption peak Vas (COO^−^) is in the range of 1,315–1,317 cm^−1^. For pure COD crystal, the Vas (COO^−^) is at 1,644 cm^−1^, while the Vs (COO^−^) is in the range of 1,324–1,325 cm^−1^. In the case of a mixture of COM and COD crystal, the positions of the asymmetric stretching vibration absorption peak and the symmetric stretching vibration absorption peak depended on the proportion of the two components in the mixture. The crystals in the UP and CA-L groups were mainly COM crystals. Notably, the Vs (COO^−^) value of the CA-L group was higher than that of the UP group. The crystals in the CA-H and CA-M groups were mainly COD crystals. This further confirmed that CA had the ability to inhibit the formation of COM crystals. Moreover, the CA - H and CA - M had the capacity to promote the formation of COD crystals.

**TABLE 4:** FT-IR absorption peak of CaOx crystal in two-dimensional agar gel system.

Groups	Vas(COO^−^)	Vs(COO^−^)
UP	1,616	1,316
CA-H	1,632	1,323
CA-M	1,631	1,317
CA-L	1,620	1,316

### 3.9 XRD characterization analysis

**FIGURE 5:**
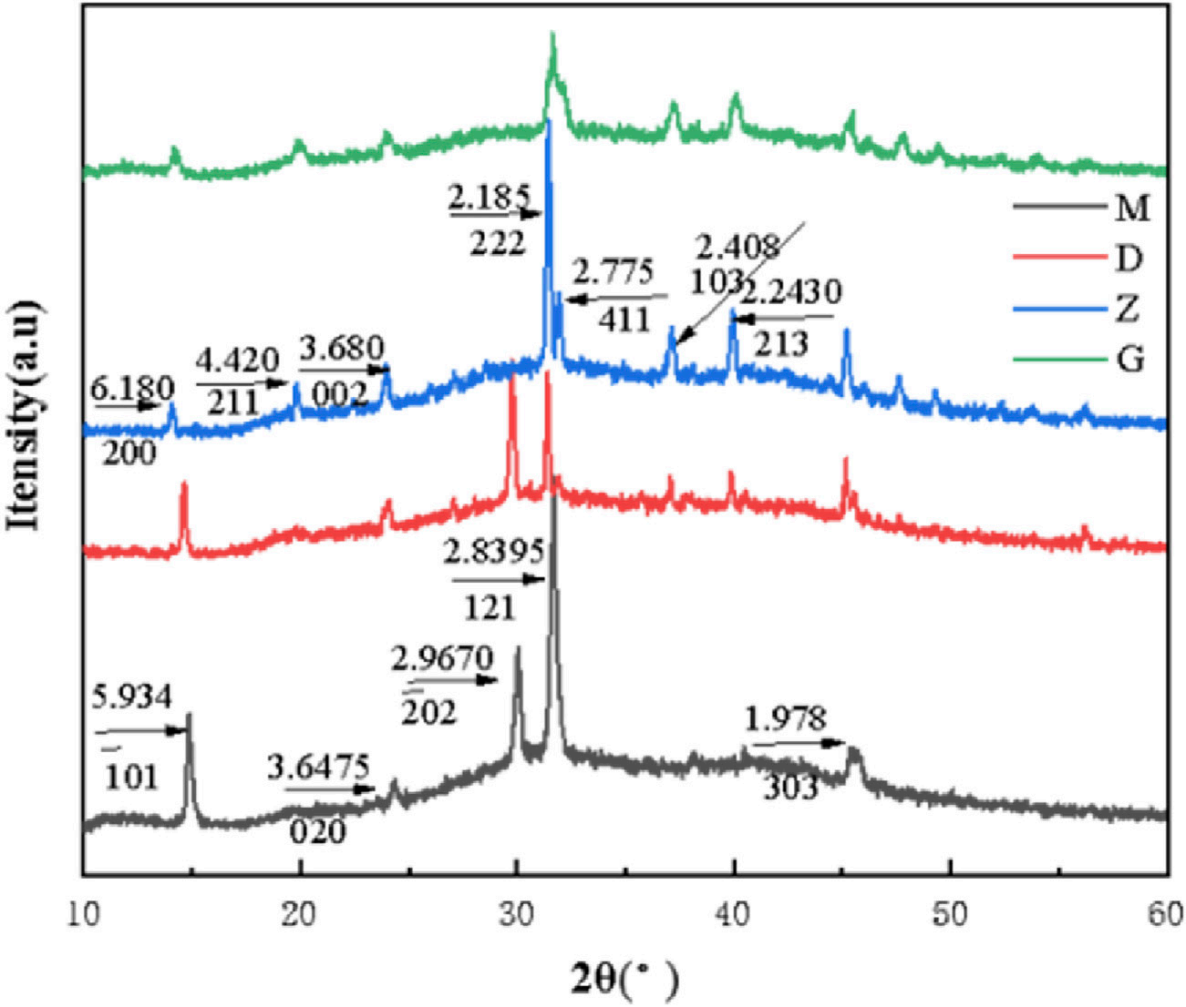
XRD spectra of CaOx crystals in two-dimensional calcium oxalate agarose gel system. (M) UP group; (D) CA-L group; (Z) CA-M group; (G) CA-H group.

The characteristic diffraction peak d values of COM crystal are 5.934, 3.6475, 2.9670, 2.8395 and 1.978, which belong to the (−101), (020), (−202), (121) and (303) crystal planes of COM crystal, respectively. The characteristic diffraction peak d values of COD crystal are 6.180, 4.420, 3.680, 2.185, 2.775, 2.408 and 2.2430, which belong to the (200), (211), (002), (222), (411), (103) and (213) crystal planes of COD crystal, respectively. The characterization results show that the characteristic diffraction peaks of COM crystals appear in both UP and CA-L groups (Figure 5), indicating that the crystals in UP and CA-L groups were mainly COM crystals; However, compared with the UP group, the absorption peak area of 2.8395 in the CA-L group was significantly smaller. The characteristic diffraction peaks of COD crystals appeared in both CA-H and CA-M groups, indicating that the crystals in CA-H and CA-M groups were mainly COD crystals, and the absorption peak area of CA-H was smaller than that of CA-M. It showed that different concentrations of CA could inhibit the formation of COM crystals, and the ability to promote the formation of COD was the strongest in CA-M, followed by CA-H, and CA-L group might only delay the aggregation of COM crystals.

### 3.10 CA alleviated the impact of COM crystal on HKC cells

**FIGURE 6:**
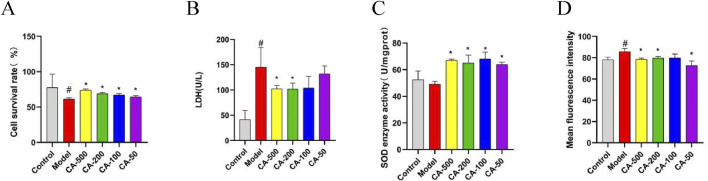
The effect of CA on HKC cell injury model induced by COM crystal. **(A)** Cell survival rate; **(B)** LDH; **(C)** SOD enzyme activity; **(D)** Mean fluorescence intensity. Compared with the normal group ^#^ *P* < 0.05, compared with the model group *P < 0.05.

To verify the effect of CA on kidney stones, an HKC cell model damaged by COM crystal was used to measure relevant indicators after CA was applied to the cells. The results indicated that, in comparison with the model group, the CA group could significantly increase cell viability (*P* < 0.05) (Figure 6A). When the concentration of CA reached 200 μg/mL or higher, the LDH leakage was significantly deccreased (*P* < 0.05) (Figure 6B), This suggested that the CA remarkably mitigated the degree of cell damage.

### 3.11 CA mitigated oxidative stress in HKC cells

To confirm the impact of CA on oxidative stress of HKC cells, the oxidative indices of cells were further measured. The results revealed that the CA group could significantly elevate the activity of SOD enzyme (*P* < 0.05) (Figure 6C), and markedly decrease the intracellular ROS level (*P* < 0.05) (Figure 6D). The results validated that the CA was effective in enhancing the antioxidant capacity of cells and alleviating the oxidative stress response within the cells.

### 3.12 CA suppressed cellular inflammatory response

**FIGURE 7:**
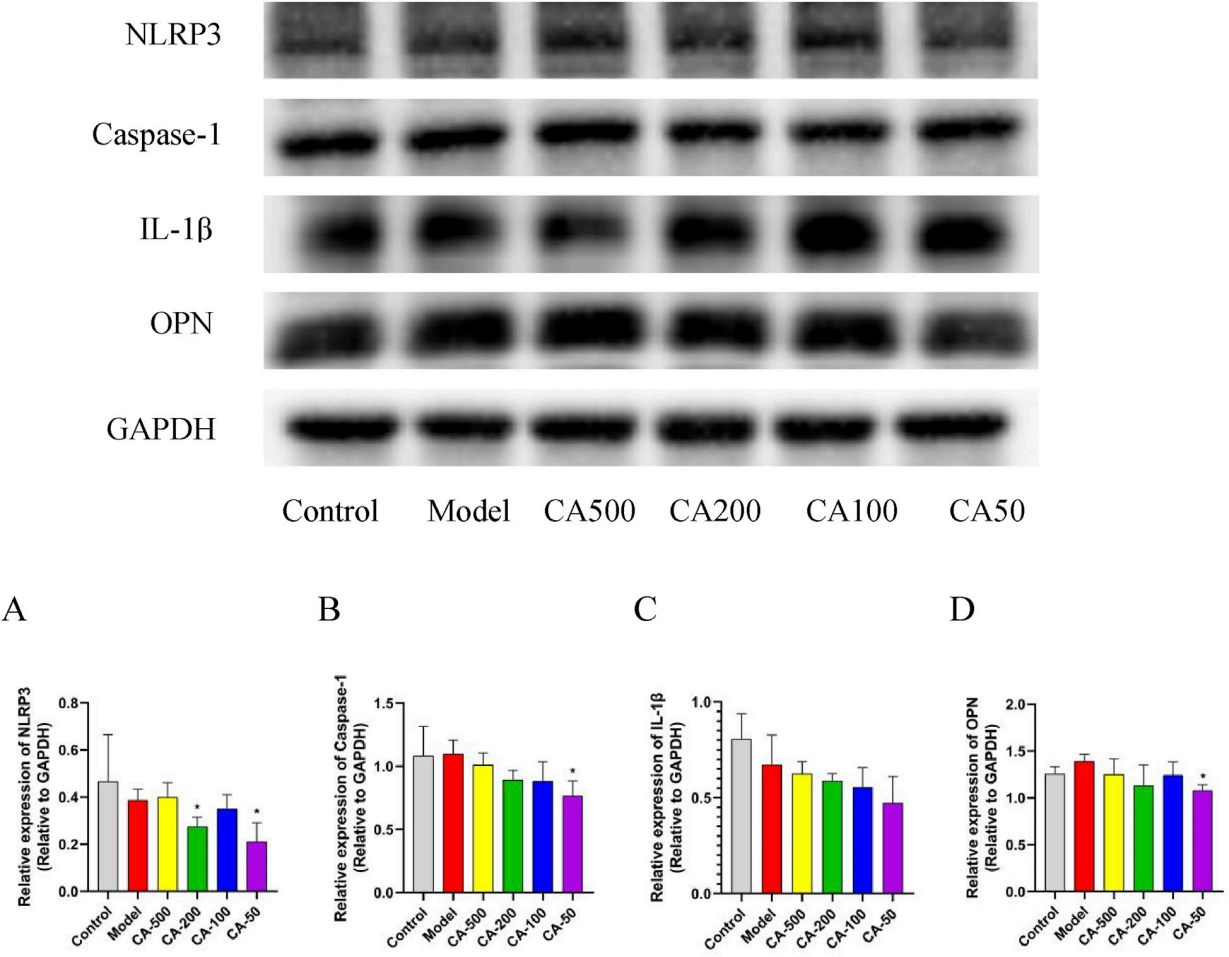
Protein expression levels in HKC cells. **(A)** NLRP3; **(B)** Caspase-1; **(C)** IL-1β; **(D)** OPN. Compared with the normal group ^#^ *P* < 0.05; compared with the model group *P < 0.05.

The impact of CA on the inflammatory response was explored by detecting the expression levels of IL-1β, Caspase-1, and NLRP3 proteins. The results showed that, in comparison with the model group, The impact of CA on the inflammatory response was explored by detecting the expression levels of IL-1β, Caspase-1, and NLRP3 proteins (*P* < 0.05) (Figure 7A). Additionally, the level of Caspase-1 in the 50 μg/mL CA group decreased significantly (*P* < 0.05) (Figure 7B). As the concentration of the CA group decreased, the expression of the IL-1β protein showed a downward trend (Figure 7C). In the drug administration groups, the expression of the OPN protein decreased with the reduction of the drug concentration. Specifically, the 50 μg/mL CA group exhibited a significant decrease in OPN protein expression compared to the model group (*P* < 0.05) (Figure 7D). These results showed that the CA might inhibit the expression of multiple targets such as IL-1β, Caspase-1, and NLRP3, thereby suppressing the cellular inflammatory response. Moreover, the downregulation of the OPN protein expression helped reduce the adhesion of crystals to damaged cells.

## 4 Discussion

Kidney stones is one of the oldest chronic diseases, which was found in ancient times. However, thousands of years later, the recurrence rate of kidney stones is still very high, and the incidence is still increasing. In the United States alone, the incidence rate has increased threefold in 3 decades, and the incidence rate of men is twice as high as that of women, and the incidence rate of women is also on the rise. The cost of treatment has increased from $ 2 billion to more than $ 10 billion over 6 years, which has placed a serious burden on the clinic and the global economy (Stamatelou et al., 2003; Scales et al., 2012; Chewcharat and Curhan, 2021). The study of kidney stones disease has been ongoing, but the pathogenesis of kidney stones is still unclear. Studies have shown that the formation of kidney stones is closely related to the inflammation of renal tubular epithelial cells damaged by calcium oxalate crystals. After cell injury, the crystal adheres to the cell, and a series of toxic reactions eventually deposit to form stones (Wigner et al., 2021). Calcium oxalate stones are the most common type of urinary stones, accounting for 80% (Khan et al., 2016). It usually exists in three forms: COM, COD and Calcium Oxalate Trihydrate (COT). COM crystals have thermodynamic stability, greater contact with renal tubular cells than COD, and have strong adhesion. It is easy to stay in the body and difficult to be excreted with urine, eventually leading to the formation of stones. The more physiological COD crystals and thermodynamically unstable COT crystals are easily excreted with urine (Ouyang, 2002). Under normal circumstances, renal tubular epithelial cells internalize or externalize calcium oxalate crystals. However, when cells are exposed to high concentrations of CaOx, they secrete a series of stress reactions such as superoxide, causing damage and even inflammation (Khan, 2004; Khan, 2014). Therefore, it is imperative to effectively reduce the damage of renal tubular epithelial cells and inhibit the adhesion of calcium oxalate crystals to avoid further aggregation and nucleation into kidney stones.

CA is a natural medicine commonly used by ethnic minorities in China. In Mongolian medicine, its preparations account for 10% and most of them are used as monarch drugs (Li et al., 2008; Zhou et al., 2017). Modern studies have shown that CA has protection of renal function, anti-inflammatory, anti-oxidation and immune enhancement (Wagner and Byrd, 2004; Di et al., 2010; Mann et al., 2020). Based on the good clinical efficacy of CA in the treatment of urinary calculi, the authors have found that the water-soluble components of CA are the effective parts of anti-calcium oxalate stones in different extracts, and can improve the formation of calcium oxalate stones in mice by reducing inflammatory immune response (Yang et al., 2010; Li et al., 2015; Zeng et al., 2020). However, its specific mechanism of action needs to be further explored. In order to clarify the mechanism of CA in the treatment of calcium oxalate stones, In this study, network pharmacology was used to predict its mechanism of action, A two-dimensional calcium oxalate agarose gel system was established for *in vitro* simulation. The generated CaOx crystals were characterized by biological microscopy, FT-IR, XRD and other methods. The effect of the aqueous extract of the CA on the growth of CaOx crystals in the two-dimensional calcium oxalate agarose gel system was discussed. And COM crystal damage HKC cell model to verify the mechanism of drug action on renal tubular epithelial cells.

Due to the complex and changeable environment in the human body, there are many factors affecting the formation of urinary stones, and the current technology is difficult to directly observe the formation process of urinary stones *in vivo*. Therefore, *in vitro* simulation method has become the first choice to study the process of stone formation. At present, the commonly used *in vitro* simulation systems include urine system, gel system and ordered molecular membrane system, Since urinary stones are usually slowly formed in the colloidal system of the urethral mucosa layer, which is similar to the crystallization precipitation phenomenon in the colloidal system, the concentric layer structure formed in the two-dimensional gel system is very close to the profile of urinary stones (Zhu et al., 2007; Zhu et al., 2011). Therefore, based on the two-dimensional calcium oxalate agarose gel system, the effect of CA on the nucleation and growth of calcium oxalate crystals was studied to clarify the mechanism of CA in the treatment of urinary calculi (Zhu, 2006). The experimental results show that in the two-dimensional calcium oxalate agarose gel system, the CA effectively inhibits the formation, aggregation and even calcification of COM crystals, promotes the formation of physiological COD crystals, and reduce the crystal face of the crystals, so that the crystals can be more easily discharged from the body.

Through network pharmacology, quercetin, kaempferol, (−)-catechin, β-sitosterol and naringenin were selected as key active ingredients, and five core targets AKT1, IL6, TNF, TP53 and IL-1β were screened out. Interleukin-6 (IL-6), as a multifunctional cytokine, is involved in the regulation of various signaling pathways, such as cell immunity, hematopoiesis, cell inflammation, cell survival, proliferation, etc. In the early stage, it is stimulated by cytokines such as TNF, which induces the differentiation of Th17 cells, inhibits the differentiation of T cells and aggravates the production of inflammatory response (Zeng and Tian, 2024). Studies have shown that reducing the level of IL-6 inflammatory factors can reduce the inflammatory response and improve the production of kidney disease (Eltahir et al., 2023). IL-1β is a classical pro-inflammatory cytokine in the IL-1 family. Usually, pro-IL-1β exists in the cell. Pro-IL-1β does not have biological activity, and activated NLRP3 is required to activate caspase-1 for protein processing and hydrolysis to exert its biological activity. It should be noted that caspase-1 can directly activate IL-1β and further activate the inflammatory mediator IL-6 in local inflammation (Wu et al., 2019). TP53 is a tumor suppressor gene. It is a complex protein with multiple functions in cell cycle. It mainly regulates cell cycle, promotes apoptosis and inhibits tumorigenesis. In immune cells, TP53 inactivation can promote the production of inflammatory cytokines and chemokines, and enhance the activity of NF-κB, promote the production of cell inflammation. TNF, as a tumor necrosis factor, binds to TNFRSF1A/TNFR1 and TNFRSF1B/TNFBR. It is mainly secreted by macrophages and can induce cell death in some tumor cell lines. It is a potent pyrogen that causes fever by direct action or stimulation of IL-1 secretion. Under certain conditions, it can stimulate cell proliferation and induce cell differentiation. AKT1 is a member of the AKT family that regulates many processes including metabolism, proliferation, cell survival, growth, and angiogenesis. AKT is a serine/threonine kinase. Activation of AKT induces nuclear translocation of NF-κB, leading to the expression of inflammatory factors in its downstream NLRP3 inflammasome, which is further involved in inflammatory diseases (Zhang et al., 2023). NLRP3 inflammasome is a defense mechanism of the body against invasion factors and its own dangerous signals.

Once the inflammasome is disordered, it will promote the secretion of inflammatory factors. Inflammasomes play a role through two steps: “initiation” and “activation”. Initiation can induce the expression of NLRP3 and pro-IL-1β by activating nuclear factor kappa B (NF-κB) through TRL, TNF, IL-1β, etc (Zhong et al., 2018). The NLRP3 inflammasome needs to be further activated after the inflammasome is initiated, and the NLRP3 inflammasome can be activated by a variety of activators. The production of mitochondrial reactive oxygen species (mtROS) is involved in the assembly of NLRP3 inflammasome, the activation of caspase-1, and the cleavage of pro-IL-1β and pro-IL-18 into mature IL-1β and IL-18. The use of ROS inhibitors can effectively inhibit the activation of NLRP3 inflammasome and reduce the production of inflammatory factors (Xu et al., 2024). These studies have shown that inhibiting the expression of NLRP3 inflammasome can reduce the expression of cellular inflammatory factors, thereby reducing cell damage and delaying the production of inflammatory diseases.

In GO analysis, the biological process mainly involves the response to oxidative stress, the regulation of inflammatory response, etc., and the molecular function mainly involves antioxidant activity, oxidoreductase activity, etc. KEGG analysis found that it was related to the NF-κB and MAPK signaling pathway. Western blot analysis showed that the expression of NLRP3 and caspase-1 in HKC cells was significantly decreased, and IL-1β decreased with the decrease of drug concentration. The activity of SOD enzyme was significantly increased and the production of ROS in cells was decreased. Therefore, CA may play a role in reducing oxidative stress injury and inflammatory response by enhancing the antioxidant capacity of cells and regulating the activation of NLRP3 inflammasome.

Osteopontin (OPN) is a secreted phosphorylated glycoprotein, which mainly participates in the inflammatory response by inducing cell adhesion, migration and inhibiting the apoptosis of inflammatory cells (Clemente et al., 2016). Over expression of osteopontin also promotes cell adhesion to crystals, inflammatory responses, and the development of various kidney diseases (Hamamoto et al., 2010; Chen et al., 2022). The OPN immobilized on collagen particles can not only promote the aggregation of calcium oxalate crystals, but also increase the adhesion of newly formed crystals (Gleberzon et al., 2019). Western blot analysis showed that the expression of OPN protein could be effectively reduced by CA, indicating that CA could reduce the adhesion of cells to calcium oxalate crystals. In addition, the CA also reduced the stimulation damage of COM crystals to HKC cells, thereby improving cell viability and avoiding the further adhesion, aggregation, nucleation, and even calcification of calcium oxalate crystals.

## 5 Conclusion

In this study, through network pharmacology analysis, it was found that the treatment of kidney stones by CA mainly involved the response to oxidative stress, the inflammatory response and the NF-κB and MAPK signaling pathway. The experimental results show that the CA can reduce oxidative stress damage, inflammatory response, and reduce the cell damage and adhesion of COM crystals by enhancing cellular antioxidant capacity, regulating NLRP3 inflammasome activation, and reducing the expression of crystal adhesion protein OPN through multiple pathways, thus avoiding further aggregation or mineralization of calcium oxalate crystals and achieving the goal of treating kidney stones. The mechanism of CA in the treatment of kidney stones was preliminarily clarified, which provided a reference for clinical application.

## Data Availability

The original contributions presented in the study are included in the article/supplementary material, further inquiries can be directed to the corresponding authors.

## Abbreviations

AKT1, AKT serine/threonine kinase 1; BP, Biological Processes; CC, Cellular Components; CA, *Choerospondias axillaris*; CaOx, Calcium Oxalate; COM, Calcium Oxalate Monohydrate; COD, Calcium Oxalate Dihydrate; COT, Calcium Oxalate Trihydrate; DL, Drug-Likeness; XRD, X-ray Diffraction; KEGG, Kyoto Encyclopedia of Genes and Genomes; FT-IR, Fourier Transform Infrared Spectroscopy; GO, Gene Ontology; HKC, Human Kidney Cortex; IL-6, Interleukin-6; IL-1β, Interleukin-1β; MF, Molecular Functions; MAPK, Mitogen-Activated Protein Kinase; NF-κB, Nuclear Factor Kappa B; NLRP3, NACHT, LRR and PYD domains-containing protein 3; OB, Oral Bioavailability; OPN, Osteopontin; PPI, Protein-Protein Interaction; TP53, Cellular Tumor Antigen p53; TNF, Tumor Necrosis Factor.
